# Extended Infusion of Dexmedetomidine to an Infant at Sixty Times the Intended Rate

**DOI:** 10.1155/2010/825079

**Published:** 2010-09-08

**Authors:** Bryan A. Max, Keira P. Mason

**Affiliations:** ^1^Department of Anesthesia, Brigham and Women's Hospital, 75 Francis Street, Boston, MA 02115, USA; ^2^Department of Anesthesia, Perioperative, and Pain Medicine, Children's Hospital Boston, Harvard Medical School, 300 Longwood Avenue, Boston, MA 02115, USA

## Abstract

Dexmedetomidine is an *α*2 adrenergic agonist which has recently been approved in the United States for procedural sedation in adults. This report describes an infant who inadvertently received an intravenous infusion of dexmedetomidine at a rate which was 60 times greater than intended. We describe the hemodynamic, respiratory, and sedative effects of this overdose.

## 1. Introduction

Infants and children frequently require sedation in order to ensure motionless conditions for radiological imaging studies. At our institution, dexmedetomidine (Precedex; Hospira, Lake Forest, IL) is the standard sedative, approved by the Hospital Sedation Committee and Pharmacy and therapeutics Committee for MRI studies. Dexmedetomidine is a highly selective *α*2 adrenoceptor agonist that possesses both sedative and analgesic effects [1]. Recently approved by the Food and Drug Administration (FDA) for procedural sedation, dexmedetomidine approval is still limited to adults only. In children, when dexmedetomidine is used as a sole agent for sedation, the doses needed to achieve adequate sedation have been shown to be remarkably high and exceed those approved for use by the FDA [2]. 

The potential hemodynamic effects of dexmedetomidine, notably sympatholysis due to *α*2 agonism at sympathetic ganglia, have been well described in healthy adults [3]. Bradycardia, hypotension, and the potential for hypertension have all been described when dexmedetomidine is administered to adults [4, 5]. The hemodynamic effects of dexmedetomidine in children, particularly when administered in greater than approved dosages, still remain to be clearly defined. Some series have reported that at higher dosages there is bradycardia and a tendency towards blood pressure variability [2, 6, 7]. The dosages required to accomplish MRI sedation with dexmedetomidine range from 2 to 3 mc/kg bolus and an infusion of 1-2 mcg/kg/hr [2, 6, 8].

The only case report in the literature of a dexmedetomidine overdosage in a child describes an elevated blood pressure and an extended recovery period [9]. Our case report describes a different sedative and hemodynamic response when an infant received an inadvertent administration of dexmedetomidine at an infusion rate of 60 times that prescribed for 20 minutes.

## 2. Case Report

A 21-month-old female with recent history of two febrile seizures (30 minutes apart) presented for an outpatient MR imaging study of the brain to complete the neurological evaluation. The infant was an otherwise healthy, full-term baby, and with an unremarkable medical history and review of systems. Upon presentation, the patient was not taking any medications although her mother carried Diastat (Acudial. Diazepam, Valeant Pharmaceuticals. Costa Mesa, CA) in the event the seizure recurred. The 9.9 kg infant presented in a calm, nonagitated state with a heart rate (HR) of 110 beats/minute, respiratory rate (RR) of 20 breaths/minute, and noninvasive brachial blood pressure (NIBP) measurement of 85/60 with a mean arterial pressure (MAP) of 68 and a room air oxygen saturation of 100%. 

**Figure 1:**
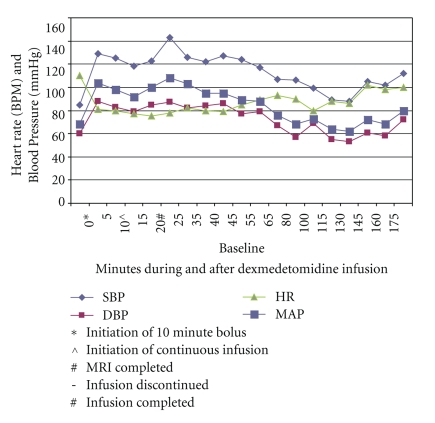


After careful review of the infant, discussion with the mother, and a physical examination, the dexmedetomidine was ordered per protocol by the pediatric nurse practitioner under the supervision of an anesthesiologist. Written, informed consent was obtained from the mother for the dexmedetomidine sedation. A 24-gauge intravenous catheter was initiated and dexmedetomidine was ordered per protocol, specifying a bolus of 2 mcg/kg over 10 minutes and a subsequent infusion of 1 mcg/kg/hr. The bolus was administered at the ordered rate of 2 mcg/kg over a period of 10 minutes using an Iradimed 3850 mRidium MR IV pump (Iradimed Corp, Winter Park, Fla). Vital signs (NIBP, MAP, 3-lead EKG, pulse oximeter, and HR) were monitored continuously using an Invivo Precess monitor (Invivo Corp, Orlando, and Fla) and documented every five minutes. The patient achieved successful sedation conditions (Ramsay Sedation Score = 4) by the completion of the bolus. Upon completion of this 10-minute bolus, NIBP was 118/79 (MAP 92), HR 77 (normal sinus), RR 17, and oxygen saturation of 97% on 2 liters/min oxygen via nasal canula (see Figure 1). Sedation guidelines at our institution specify supplemental oxygen delivery throughout the sedation and recovery period until discharge criteria are met. Following the bolus, the infusion pump initiated delivery of the dexmedetomidine infusion at the rate that had been programmed in at the initiation of the sedation. The dexmedetomidine infusion was continued throughout the sedation until the MRI scan was complete. The patient remained hemodynamically stable throughout Vital signs were documented at five-minute intervals per our hospital standard for sedation, following initiation of this dexmedetomidine infusion (Table 1). 

At the termination of the study, the radiology nurse noted that the dexmedetomidine remaining in the syringe was less than anticipated and medication reconciliation was immediately initiated with a second nurse. Reconciliation revealed that the 9.9-kg infant had received 196 mcg more dexmedetomidine than had been ordered. Review of the intravenous infusion pump revealed that the pump had been misprogrammed to deliver the infusion at a rate of mcg/kg/min rather than mcg/kg/hr. Thus, instead of delivering the usual 1 mcg/kg/hour as ordered, the infusion pump delivered the dose at 1 mcg/kg/minute (equivalent to 60 mcg/kg/hr). Because this error was not identified during the mandatory institutional nursing “double check” when the infusion pump was programmed, the error was not identified, and the dexmedetomidine infusion was continued for the 20 minutes. 

Following the imaging study, the patient was transferred uneventfully to the radiology recovery room at which time monitoring was continued. The patient arrived in the recovery room deeply sedated with an RSS 4. Per recovery room policy, NIBP, MAP, EKG, HR, O2 Sat, and RR are documented every 5 minutes until modified Aldrete discharge criteria are met [10]. A minimum Aldrete score for discharge from the recovery room is 9. Although documented every 5 minutes, pulse oximetry is monitored continuously. Throughout the recovery room course, all physiologic parameters remained within age-adjusted normal values. 20 minutes after the discontinuation of the dexmedetomidine, the infant had achieved a modified Aldrete score of 9 and maintained an Aldrete of 9-10 throughout the remainder of the recovery room stay. No cardiac arrhythmias were noted at any time, both during the sedation as well as in the recovery room period. As soon as the error in dosing was identified, Risk Management was notified and the parents were debriefed and all questions answered by the Risk Management team as well as the supervising anesthesiologist. The parents were informed that the intravenous infusion pump had been misprogrammed and that the child had received an infusion of 60 times that which was ordered and intended. The effects of such a high dosage of dexmedetomidine delivery to an infant had not to date been described nor documented. In adults, the distribution half-life (t1/2) is approximately 6 minutes and the terminal elimination half-life (t1/2) is approximately 2 hours [11]. 

The infant returned to baseline neurological status, an Aldrete Score of 10, after a 2-hour recovery room period. Although the half-life of dexmedetomidine is relatively short, and the child met discharge criteria with a modified Aldrete Score of 10 within hours of discontinuing the dexmedetomidine, the physicians chose to admit her to the hospital for overnight, continuous cardiorespiratory monitoring. Subsequently, the patient was admitted to the intensive care unit (ICU) for overnight monitoring of EKG as well as pulse oximetry, blood pressure, and neurological status. The child remained hemodynamically and neurologically stable in the ICU throughout the night. There were no arrhythmias, no change in neurologic status nor any deviation in heart rate or blood pressure outside of age-adjusted anticipated normal values. The next morning, after reassessment by neurology and the intensive care unit service, the infant was discharged home with no subsequent sequela.

## 3. Discussion

Dexmedetomidine (Precedex) is a relatively selective *α*2-adrenergic agonist with sedative properties. As an imidazole, dexmedetomidine has an *α*2 : *α*1 activity ratio of 1620 : 1, compared to 220 : 1 with clonidine [12]. It is known that spinal and supraspinal *α*2-adrenoreceptors mediate and modulate nociception. These receptors are widely distributed throughout the peripheral and central nervous systems and a variety of organs, including liver, kidney, and pancreas. *α*2-adrenoreceptors have been located at presynaptic, postsynaptic, and extrasynaptic sites. Of these, the presynaptic and postsynaptic receptors may be the more clinically important in analgesia. In general, activation of *α*2-presynaptic receptors inhibits norepinephrine release and possibly substance P release, thereby inhibiting pain signal transmission. Postsynaptic activation in the central nervous system inhibits sympathetic activity, thus moderating heart rate and blood pressure [13–18]. Together, these effects produce analgesia, sedation, and anxiolysis.

Recently approved (October 2008) for procedural sedation outside of the intensive care unit setting, Dexmedetomidine is still not approved by the Food and Drug Administration (FDA) for pediatric use. Although not approved in children, its use has been described for pediatric sedation in the intensive care unit, for radiology, gastroenterology, and dental procedures, as well as for electroencephalograms [19–28]. The dosages required to achieve sedation in infants and children tend to be higher than for adults. The need for higher dosages in children as compared to adults is confirmed in a recent pharmacokinetic study [29]. 

 In adults, dexmedetomidine can produce varying depths of sedation which have been compared to states of natural sleep with respect to cardiovascular and respiratory effects [1]. When administered to adults within clinical dosing guidelines, there are no demonstrated significant accompanying changes in resting ventilation [3, 30, 31]. In fact, there is an evidence to support that dexmedetomidine actually mimics some aspects of natural sleep in both children and adults [30, 32]. 

Our concern with this infant following the overdosage was the potential for hemodynamic compromise. In both adults and children, there may be an increased incidence of clinically significant bradycardia with hypotension and possibly even cardiac arrest with dexmedetomidine, especially when administered with other medications which possess negative inotropic or chronotropic effects [33]. The potential hemodynamic effects of dexmedetomidine, notably sympatholysis, have shown a biphasic physiologic response characterized by an initial increase in systolic blood pressure and a reflex-induced decrease in heart rate followed by stabilization of heart rate and blood pressure below baseline values. The initial increase in MAP reportedly lasts for 5–10 minutes with a subsequent decrease in MAP of approximately 10%–20% below baseline with an HR that usually stabilizes below baseline values. These effects have been attributed to an inhibition of the central sympathetic outflow [30, 34]. Hemodynamic variability with dexmedetomidine has been described in case reports of severe bradycardia in a child with digoxin, hypertension in a child with traumatic brain injury, and hypertension in a child with acute transverse myelitis [5, 35, 36]. Children who received dexmedetomidine (3 mcg/kg bolus and 2 mcg/kg infusion) are more likely to manifest hypertension if they are less than one year of age and have received more than one bolus of dexmedetomidine [7]. 

At our institution, radiology sedation is administered by nurses under the direct supervision of a pediatric nurse practitioner and supervising anesthesiologist. All children must be medically appropriate to receive dexmedetomidine sedation and cannot have any conditions which our institutional Hospital Sedation Committee considers to be a contraindication to dexmedetomidine [2, 6, 8] (Table 2). Dexmedetomidine sedation is administered following protocols which are on preprinted, templated order sheets approved by the Pharmacy and Therapeutics Committee. The order sheet specifies the following. A bolus of dexmedetomidine is administered at a specified dose in mcg/kg over 10 minutes. The goal of the bolus is to achieve a minimum Ramsay Sedation Score (RSS) 4 [37]. An RSS 4 or RSS 5 is a clinically derived scoring system that is generally accepted as the depth of sedation adequate to facilitate diagnostic imaging studies [8, 38]. This bolus may be repeated at the same dosage and time interval if the patient fails to achieve or maintain the minimum RSS 4, at any time during the sedation. Following completion of the bolus, and confirmation of adequate sedation, an infusion at a specified dosage in mcg/kg/hr is immediately started and continued until completion of the study. Following completion of the MR scan, the dexmedetomidine is discontinued, the patient is transported to the radiology recovery room and monitored with documentation of vital signs every 5 minutes until discharge criteria are met. Per our institutional guidelines, discharge criteria require a minimum Aldrete Score of 9 points [10].

In summary, this report describes the outcome during and following a substantial, inadvertent overdosage of dexmedetomidine to an infant. The overdosage represents a continuous dexmedetomidine infusion for 20 minutes at a rate which was almost 80 times that was recommended by the Food and Drug Administration for adults. To date, most of the reports of inadvertent overdosage are in adults, with the degree of overmedication substantially less than cited in our report [39]. The child received 60 mcg/kg/hour for 20 minutes and maintained cardiovascular and respiratory stability. Our experience differs from a previous overdose report which described an increase in blood pressure [9]. The absence of a significant hypertensive response suggests that infants may not demonstrate the biphasic hemodynamic response for blood pressure and vascular resistance as is reported in the adult literature [40]. This report is important because it demonstrates that even in excessive dosages, dexmedetomidine may not elicit an extreme hemodynamic or respiratory effect. This child exhibited a prolonged recovery and somnolence, with almost 2 hours to meet Aldrete discharge criteria. This prolonged recovery period is longer than the average 30 minutes recovery time, when the prescribed dosage is administered [2]. 

Although the infant in this report did not suffer any noticeable short- or long-term adverse sequela, our experience, however, identifies a serious and important mishap; Dexmedetomidine is unusual in that it is administered as an infusion with an hourly rate rather than a rate expressed per minutes. Both nursing and physicians are more commonly habituated to administer infusions per minute. Thus, careful double checks of the programmed rate must be followed in order to ensure accurate delivery of dexmedetomidine. There are no clear guidelines from the Joint Commission for the programming and delivery of intravenous medication. Rather, standard practice in our institution requires that the accurate programming of the infusion pump be independently verified by two separate nurses. Even with this process in place and documentation by two separate nurses that each had independently performed and verified the drug concentration, bolus and infusion dosage, and rate of administration, the error occurred. As a result of this incident, our institution has added an additional safety measure. The dexmedetomidine program in the infusion pump has been restricted to deliver infusions in units of mcg/kg/hour and a bolus in units of mcg/kg. The ability to deliver dexmedetomidine at a mcg/kg/minute rate or mg/kg dosage has been lockedout.

In conclusion, despite a large overdose of dexmedetomidine, this infant demonstrated hemodynamic stability throughout without incidence of hypotension or hypertension. Aside from the prolonged sedation for up to 2 hours following discontinuation of the infusion, she suffered no additional sequela. This report reveals the need for continued study of dexmedetomidine in order to determine the optimal dosing to ensure successful sedation conditions, hemodynamic stability, and a safe recovery period.

## Figures and Tables

**Table 1:** Vital Signs at time points (minutes) prior to, during, and following initiation of the dexmedetomidine bolus and infusion.

	Presedation/Baseline	0	5	10	15	20	25	30	35	40	45
NIBP	85/60	118/79	123/85	143/87	126/82	122/84	127/86	124/72	117/79	107/67	106/57
MAP (mmHg)	68	92	100	108	103	95	95	89	88	76	68
Heart Rate (BPM)	110	77	75	78	82	85	79	85	89	93	90
RR (breaths/min)	20	17	13	24	13	14	14	16	18	18	18
O2 Saturation	100%	97%	96%	96%	96%	97%	97%	98%	98%	99%	99%
EKG	NSR	NSR	NSR	NSR	NSR	NSR	NSR	NSR	NSR	NSR	NSR

NSR: Normal Sinus Rhythm

MAP: noninvasive mean arterial blood pressure

RR: Respiratory rate

0–10 minutes: The dexmedetomidine bolus administered

10–20 minutes: The dexmedetomidine infusion initiated and completed

20–45 minutes: The recovery room period until discharge criteria, defined as a minimum modified Aldrete Score 9, are achieved.

**Table 2:** Medical Conditions Which Contraindicate Dexmedetomidine Sedation.

Active, uncontrolled gastroesophageal reflux—an aspiration risk
Active, uncontrolled vomiting—an aspiration risk
Current (or within past 3 months) history of apnea requiring an apnea monitor
Active, current respiratory issues that are different from the baseline status (pneumonia, exacerbation of asthma, bronchiolitis, and respiratory synctitial virus)
Unstable cardiac status (life threatening arrhythmias, abnormal cardiac anatomy, and significant cardiac dysfunction)
Craniofacial anomaly, which could make it difficult to effectively establish a mask airway for positive pressure ventilation if needed
Current use of digoxin
Uncontrolled hypertension
Moya Moya Disease
New-onset stroke
